# Exploration of radiotherapy strategy for brain metastasis patients with driver gene positivity in lung cancer

**DOI:** 10.7150/jca.91875

**Published:** 2024-02-12

**Authors:** Qian Bi, Xin Lian, Jing Shen, Fuquan Zhang, Tao Xu

**Affiliations:** 1 Department of Radiation Oncology, State Key Laboratory of Complex Severe and Rare Diseases, Peking Union Medical College Hospital, Chinese Academy of Medical Science and Peking Union Medical College, Beijing, China.; 2 Department of Epidemiology and Biostatistics Institute of Basic Medical Sciences, Chinese Academy of Medical Sciences & School of Basic Medicine, Peking Union Medical College.

**Keywords:** brain metastases, radiation therapy, lung cancer with brain metastasis, WBRT

## Abstract

**Objective:** To assess the disparities in effectiveness and identify outcome predictors in the treatment of a targeted-first and radiotherapy-first regimen with driver gene-positive lung cancer brain metastases.

**Materials and Methods:** This retrospective study analyzed patients with driver gene-positive lung cancer brain metastases who received first-targeted and first-radiotherapy regimens, respectively, with SIB-WBRT (whole brain tissue 40 Gy/20 fractions, tumor tissue boosted to 56-60 Gy/20 fractions) and local irradiation (prescription dose range of 20-60 Gy/2-25 fractions, most commonly delivered as 30 Gy/5 fractions, with a BED range of 28-100.8 Gy) at Peking Union Medical College Hospital from September 2015 to December 2021. The primary endpoint was intracranial progression free survival (iPFS). Secondary endpoints included overall survival (OS), intracranial new lesions, and tumor control. The Kaplan-Meier method was utilized to depict and estimate iPFS, OS, intracranial new lesions and tumor control. The Cox regression analysis was conducted to assess the association between relevant factors and outcomes.

**Results:** 88 patients were enrolled in targeted-first and radiotherapy-first regimen, totally. And no difference was found in the comparison of iPFS between the two groups (HR=1.180, 95%CI: 0.622-2.237, *P*=0.613). No difference was found in the comparison of OS between the two groups (HR=1.208, 95%CI: 0.679-2.150, *P*=0.520). No difference was found in the comparison of intracranial new lesions between the two groups (HR=1.184, 95%CI: 0.569-2.463, *P*=0.652). There was a difference in the local control time between the two groups, with radiotherapy-first regimen being superior (HR=2.397, 95% CI:1.453-3.954, *P*<0.001). Patient age (HR=1.054, 95%CI: 1.026- 1.082, *P*<0.001), radiotherapy modality (HR=0.128, 95%CI: 0.041-0.401, *P*<0.001), metastasis volume (HR=1.426, 95%CI: 1.209-1.682, *P*<0.001), number of metastases(HR=14.960, 95%CI: 1.990-112.444, *P*=0.009), extracranial disease status (HR=0.387, 95%CI: 0.170-0.880, *P*=0.023) and therapy sequence (HR=13.800, 95%CI: 4.455-42.751, *P*<0.001) were associated with local control.

**Conclusion:** Targeted-first regimen was not found to improve patients' iPFS relative to radiotherapy-first regimen in patients with brain metastases. Radiotherapy-first regimen for brain metastases demonstrated superior local control compared to targeted-first regimen. Patient's age, radiotherapy modality, metastasis volume, number of metastases, extracranial disease status and therapy sequence may be related to local control of metastases.

## Introduction

In recent years, with the prolonged survival of lung cancer patients, brain metastasis has become a serious clinical issue that has received increasing attention 1. This disease not only poses a significant threat to patients' survival but also causes serious interference to their quality of life. Lung cancer patients with brain metastasis often face multiple challenges such as neurological dysfunction, which severely limits their daily activity and life satisfaction 2. Therefore, in order to improve the survival status and quality of life of lung cancer patients with brain metastasis, it is urgent to explore effective treatment strategies. Currently, radiotherapy is one of the important treatment modalities for brain metastatic tumors, and targeted therapy also plays an increasingly important role.

Radiotherapy is one of the three traditional cancer treatment methods, which utilizes high-energy radiation to irradiate tumor tissue and disrupt the DNA double helix structure for therapeutic purposes. However, radiotherapy also has shortcomings. Firstly, during the radiotherapy process, normal brain tissue is inevitably exposed to radiation, which may lead to adverse reactions such as cognitive dysfunction and neurological toxicity. Additionally, radiotherapy is a complex, time-consuming, and labor-intensive process, which places a significant burden on the current limited medical resources 3. With the progress of basic research, targeted therapy has gradually gained popularity as a new treatment strategy. Targeted therapy drugs target specific molecular targets in malignant tumors, aiming to inhibit tumor growth and spread by blocking signal pathways. This treatment accurately targets specific molecular targets, avoiding unnecessary damage to normal tissue, and does not cause adverse reactions such as neurological toxicity and cognitive dysfunction, greatly improving patients' quality of life. However, there is no consensus in the academic community regarding whether to prioritize targeted therapy or radiotherapy when determining the treatment plan for lung cancer brain metastasis patients with driver gene mutations.

Therefore, we retrospectively analyzed the survival status of patients who received either radiotherapy-first or targeted-first therapy at our hospital during the entire course of brain metastasis, aiming to provide some empirical support for the advantages and disadvantages of these two treatment modalities. Additionally, we compared our results with similar studies published internationally.

## Methods

### Patient enrollment

This study was a retrospective study. The study subjects were lung cancer brain metastases patients with driver gene-positive who received SIB-WBRT and brain metastases local irradiation treatment at the Department of Radiation Oncology, Peking Union Medical College Hospital from September 2015 to December 2021. Patients' inclusion criteria were:

(1) pathologically confirmed lung cancer and confirmed brain metastases by imaging;

(2) brain metastasis radiotherapy using SIB-WBRT or brain metastases local irradiation;

(3) completion of the entire course of radiotherapy;

(4) at least one post-treatment imaging examination available for evaluating treatment efficacy. Patients' exclusion criteria were:

(1) no follow-up results after treatment.

This study has been approved by the Ethics Review Committee of Peking Union Medical College Hospital (Approval number: S-K1982), and informed consent has been obtained from all subjects.

### Data collection

Relevant data of patients were obtained from the electronic medical record system of Peking Union Medical College Hospital. The following data were collected:

(1) clinical data: including primary disease, pathological type, molecular pathology, extracranial disease progression, number and volume of metastatic tumors, radiotherapy technique and dose fractionation, concurrent medication, etc.;

(2) general data: including general information such as gender, age, etc.;

(3) follow-up data: including treatment response of intracranial lesions (lesion control and appearance of new lesions), survival status, follow-up time, etc.

### Radiation therapy method

6MV X-ray linear accelerator was used for radiation therapy including volumetric modulated arc therapy (VMAT) technique, fixed field-intensity modulated radiotherapy (FF-IMRT) technique or TOMO Therapy (Tomo) technique. Thermoplastic mesh was used for patient immobilization, and 2-3mm CT slices were used for simulation and positioning. The positioning image was fused with 3D-T1 enhanced MRI for delineation of target areas and organs at risk (OARs). The clinical target volume (CTV) was defined as the entire brain tissue, and the gross tumor volume (GTV) was defined as the visible tumor area on MRI image. Both CTV and GTV were expanded by 2-3mm to form the planning target volume (PTV). The prescribed dose at the GTV was 56-60Gy (median of 60Gy), and treatment plans were designed using Tomo, Eclipse, or Monaco treatment planning systems. The prescribed dose at the CTV was 40Gy, and both were completed within 20 fractions, with 5 treatments per week. The volume dose limits for OARs were: ≤8Gy for 1% of the lens, and ≤54Gy for 0.03cc of the brainstem.

When brain metastases local irradiation is used, the gross tumor volume (GTV) was defined as the visible tumor area on MRI images. GTV were expanded by 2-3mm to form the planning target volume (PTV). The GTV was prescribed in a dose range of 20-60 Gy (number of fractions ranging from 2-25), with the most common fractionation pattern being 30 Gy/5 fractions, and the BED of the whole group of patients ranging from 28-100.8 Gy.

### Patient follow-up

All patients were followed up one month after treatment, then after that outpatients would be followed up every 3 months. Enhanced MRI of the head is used for assessment of the therapeutic efficacy of brain metastases radiotherapy, based on the Response Assessment in Neuro-Oncology (RANO) criteria, by experienced radiation oncologists 4. Safety assessment was performed in the entire cohort, including radiation necrosis, hematology, and biochemical parameters. Adverse events were graded according to the Common Terminology Criteria for Adverse Events (Version 5.0) of the National Cancer Institute 5.

### Follow-up endpoints

The primary endpoint was intracranial progression-free survival (iPFS), defined as the time from the end of radiotherapy to intracranial radiographic progression or death 6. Radiographic progression includes uncontrolled recurrence of lesions or the appearance of new intracranial lesions 7.

Secondary endpoints included overall survival (OS), tumor local control time, and time to new intracranial lesions. OS was defined as the time from the end of radiotherapy to death or the last follow-up 8. Tumor local control time was defined as the time from the end of radiotherapy to the detection of tumor recurrence or the last follow-up. Time to new intracranial lesions was defined as the time from the end of radiotherapy to the detection of new lesions or the last follow-up.

### Statistical analysis

Based on chart review, 88 patients met the inclusion criteria. A total of 44 patients were followed up in this study for the primary outcome event, with a ratio of 56 patients to 32 patients in the intervention and control groups, and assuming a bilateral alpha of 0.05, this sample size has a treatment effect with 80% certainty of finding a HR of less than 0.415. Kaplan-Meier method was used to depict and estimate iPFS, OS, incidence of new lesions, and tumor control. If a patient had multiple lesions, multiple lesions data were analyzed as data basic points for local control. For the primary research objective of this study, to compare the prognosis of patients in the targeted-first and radiotherapy-first regimen groups of brain metastases, a Cox regression model was constructed in which the dependent variable was the prognostic outcome of the patients, and the main explanatory variables were the grouping factors of targeted-first and radiotherapy-first regimen. Effect sizes for the comparison of treatment outcomes between the two groups were reported using hazard ratios (HR) and reported with 95% confidence intervals (CI). Cox model was used to analyze the correlation between relevant factors (gender, age, pathological type of lung cancer, tumor volume, number of tumors, control of primary lesions during brain metastases, targeted therapy during the course of brain metastases, and proportion of tumors in the whole brain) and outcomes, and both univariable and multivariable analyses were conducted. All factors were included in the multivariable analysis regardless of statistical significance in the univariable analysis. All statistical analyses were performed using SPSS version 27.0 (IBM Corp, New York, USA), GraphPad Prism 8, and SAS 9.4 (SAS Institute Inc, Cary, NC). The significance level was set at a two-sided *P*-value of <0.05^9^.

## Results

### Patient baseline clinical characteristics

A total of 88 patients with 427 brain metastases were included in this study according to the inclusion criteria. Among them, 32 patients received targeted therapy first, and 56 patients received radiotherapy first. The general characteristics of the patients are shown in Table 3-1,^9^. The number of metastatic tumors in the group treated with targeted therapy first was relatively small, with only 1 case (3.1%) having more than 10 metastases, and there were fewer patients with uncontrolled primary tumors 31(96.9%). The two groups had similar characteristics such as age, gender, receipt of targeted therapy, and volume of metastatic tumor involvement in the entire brain tissue. In the overall population, there were 88 patients with non-small cell lung cancer, including 51 females and 37 males. All 88 patients received targeted therapy during the entire brain metastasis course. Three patients had well-controlled primary tumors during radiotherapy of the brain, while the other 85 patients had poorly controlled primary tumors during radiotherapy of the brain.

The median value of the radiation treatment planning system estimated the tumor volume at the time of enrollment as 5.6 cm^3^ (0.3-81.6 cm^3^). There were 80 patients with less than 10 brain metastases and 8 patients with more than 10 brain metastases. The median value of the average radiation dose to the brain was 41.3 Gy (0.621-46.13 Gy). The median value of the metastatic tumor proportion in the whole brain tissue was 0.504% (0.03%-5.80%) 10. The details are shown in Table 1.

Compared with radiotherapy-first, there was no significant improvement in iPFS with targeted-first therapy (HR=1.180, 95% CI: 0.622-2.237, P=0.613) 11. A total of 53.1% and 48.2% of patients in the targeted therapy and radiotherapy groups, respectively, had no intracranial progression. The 6- and 12-month intracranial progression-free survival rates were 66.1% (95% CI: 47.700-84.500) and 45.1% (95% CI: 23.700-66.500) in the targeted therapy group and 87.1% (95% CI: 77.300-96.900) and 53.5% (95% CI: 36.600-70.400) in the radiotherapy group. The median iPFS values were 13.4 (95% CI: 6.860-20.000) months and 15.6 (95% CI: 7.700-23.500) months in the targeted therapy and radiotherapy groups, respectively.

Compared with radiotherapy-first, there was no significant improvement in OS with targeted-first therapy (HR=1.208, 95% CI: 0.679-2.150, P=0.520). A total of 43.75% and 39.29% of patients in the targeted therapy and radiotherapy groups, respectively, survived. The 6- and 12-month survival rates were 80.3% (95% CI: 66.200-94.400) and 67.7% (95% CI: 49.900-85.500) in the targeted therapy group and 85.5% (95% CI: 76.100-94.900) and 70.8% (95% CI: 58.300-83.300) in the radiotherapy group. The median OS values were 17.9 months (95% CI: 13.400-26.000) and 23.5 months (95% CI: 14.700-32.300) in the targeted therapy and radiotherapy groups, respectively.

Compared with radiotherapy-first, there was no significant improvement in new lesion with targeted-first therapy (HR = 1.184, 95% CI: 0.569-2.463, P = 0.652). The incidence of new intracranial lesions was 34.38% and 42.86% in the targeted and radiotherapy first groups, respectively. The 6- and 12-month incidence rates of no new lesions were 68.6% (95% CI: 50.200-87.000) and 60.1% (95% CI: 37.600-82.600) in the targeted therapy group and 89.2% (95% CI: 80.200-98.200) and 66.5% (95% CI: 50.200-82.800) in the radiotherapy group. The median new lesion values were 17.4 months (95% CI: 2.400-32.400) and 18.1 months (95% CI: 14.400-21.700) in the targeted therapy and radiotherapy groups, respectively.

**Figure 1:**
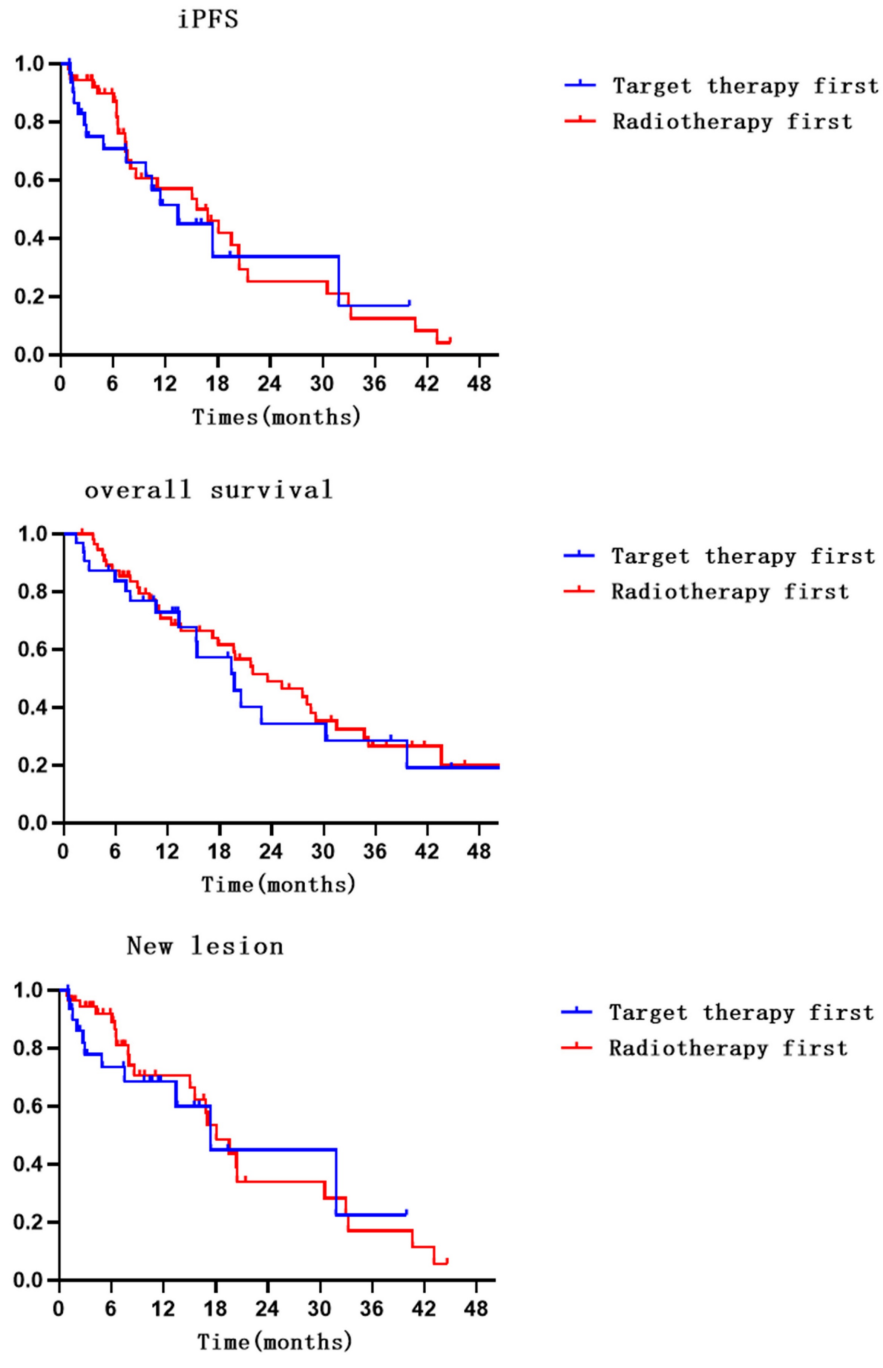

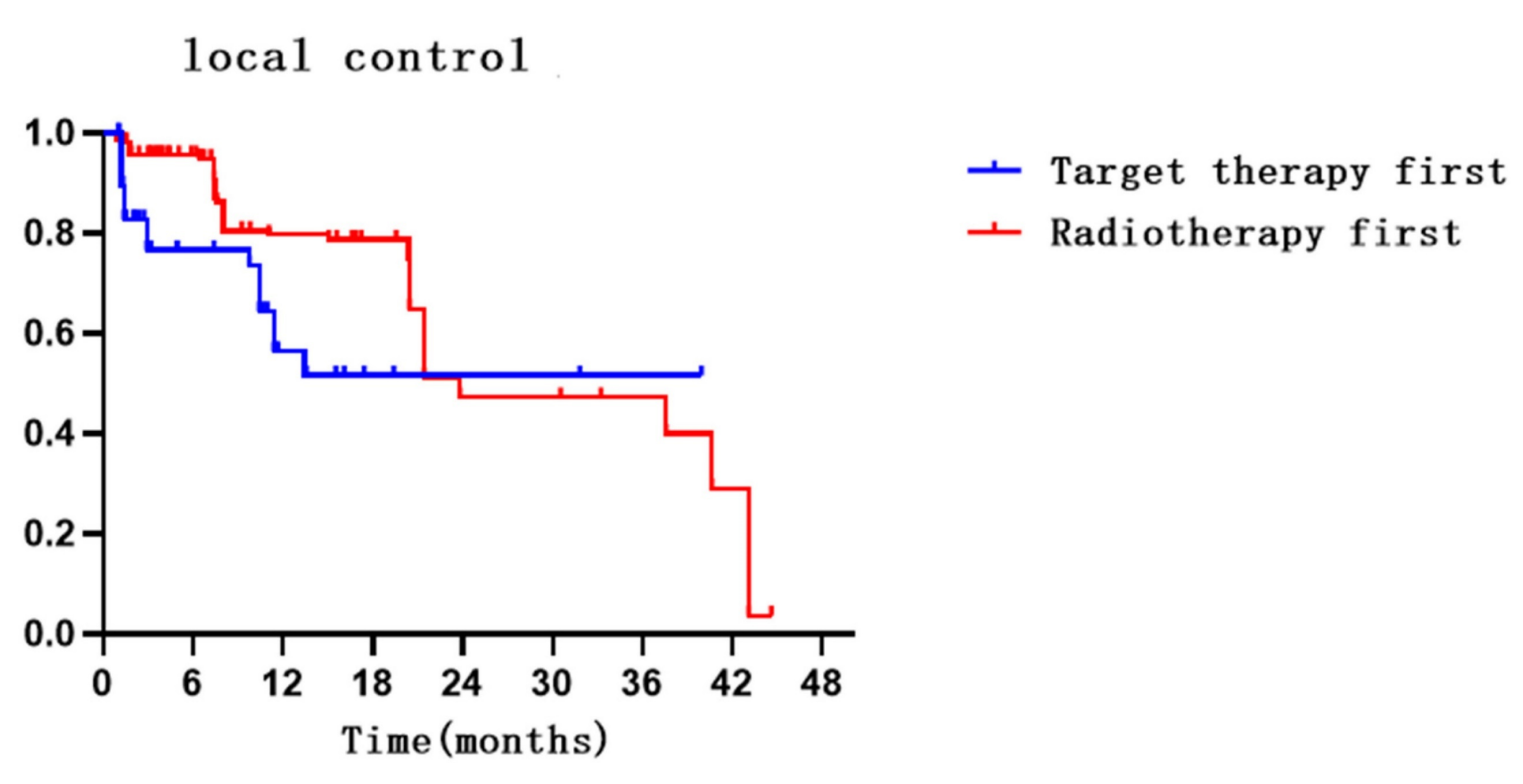
Survival, local control, iPFS, and new lesions survival curves for patients in both groups.

Compared with targeted-first therapy, radiotherapy-first can better improve local control in lesions (HR = 2.397, 95% CI: 1.453-3.954, P < 0.001). A total of 76.0% and 80.7% of lesions in the targeted therapy and radiotherapy groups, respectively, were well controlled. The 6- and 12-month intracranial control rates were 73.6% (95% CI: 62.200-84.900) and 51.6% (95% CI: 34.400-68.800) in the targeted therapy group and 94.9% (95% CI: 92.200-97.600) and 78.7% (95% CI: 72.000-85.400) in the radiotherapy group. The median local control values were NR months and 23.8 months (95% CI: 15.200-32.500) in the targeted therapy and radiotherapy groups, respectively. Details are shown in Table 2, Supplementary Table 6 and Figure 1.

### Radiation necrosis

No cases of radiation necrosis were observed in the follow-up results of this patient group.

### Univariable and multivariable analysis

Using Cox regression models to analyze associations between factors and outcomes. Regarding iPFS and new lesions, univariable analyses showed that no significant predictors of iPFS and new lesions were found. Multivariable analysis using Cox proportional risk regression modeling similarly did not identify significant predictors of new lesions and iPFS.

Regarding local control, univariable analysis showed that age, metastasis volume, extracranial disease status, therapy sequence, and number of metastases were significant predictors of local control. Multivariable analysis using Cox proportional risk regression model showed that age, radiotherapy modality, metastasis volume, number of metastases, extracranial disease status and therapy sequence were significantly associated with local control (Table 5), and in summary, age, radiotherapy modality, metastasis volume, number of metastases, extracranial disease status and therapy sequence might be independent prognostic factors for local control. For details, see Tables 3, 4, and 5.

## Discussion

The patients included in this study were from various parts of China, and the data results are more extensive and representative than those of single-center retrospective studies. The relevant data results can effectively fill the gap in this field. In this single-center retrospective study, we summarized the efficacy of the targeted-first therapy regimen and the radiotherapy-first regimen in controlling intracranial metastatic lesions in patients with brain metastases. The primary endpoint iPFS had a median value of 13.4 months (95% CI: 6.860-20.000) and 15.6 months (95% CI: 7.700-23.500) for the targeted therapy and radiotherapy groups, respectively, and there was no significant difference (HR = 1.180, 95% CI: 0.622-2.237, P = 0.613). The survival status of patients who received the two treatment regimens was similar. Regarding toxicity, no radiation necrosis was observed in all patients, indicating that the efficacy of the two modalities is safe and reliable. The radiotherapy-first regimen may be more beneficial for local control of metastatic lesions.

The sequence of targeted therapy and radiotherapy for the treatment of lung cancer brain metastasis with driver gene mutations remains controversial. Some studies have suggested that targeted-first therapy can effectively control systemic tumor progression and reduce the burden of brain metastasis, followed by consideration of local radiotherapy needs 12, 13. A study published in 2019 retrospectively analyzed 104 patients and found no significant difference in survival between the patients who received targeted-first therapy and those who did not 12. The study authors therefore recommended targeted-first therapy to preserve cognitive function, followed by rescue radiotherapy later. Other studies support radiotherapy-first to control local disease and alleviate related symptoms, followed by targeted therapy to control systemic disease 14, 15. A retrospective study conducted by Yale University in 2017 explored this issue by including data from multiple centers. After further analysis, it was found that for EGFR mutation NSCLC patients, targeted-first therapy with delayed radiotherapy was associated with poorer OS 14. The results of a study by William J Magnuson et al. were consistent with these findings, showing that targeted-first therapy with delayed radiotherapy may lead to poorer OS for EGFR mutation NSCLC patients at their center 15. However, most of the previous studies in this area were retrospective studies with low evidence levels, and further prospective, multi-center randomized studies or meta-analyses are needed for more powerful evidence to compare and validate the treatment sequences.

The targeted-first therapy and radiotherapy-first treatment sequences have not been shown to significantly differ in terms of patient survival outcomes. Recent studies have demonstrated that there is no significant difference in patient survival between the targeted-first therapy and radiotherapy-first treatment sequences in patients with EGFR mutation-positive lung cancer brain metastases 16-18. A clinical study conducted by Ke et al. in 2018 observed 10 patients in the targeted therapy alone group who received targeted therapy first and then received radiotherapy as salvage therapy for failed metastasis control, and 10 patients in the radiotherapy alone group who received radiotherapy first. The results showed that the overall survival (OS) was 39 months and 48 months, respectively, with no significant difference between the two groups (P=0.849) 16, 19. Additionally, a study conducted by Japanese scholars in 2019 compared the treatment sequences of EGFR-positive lung cancer brain metastases patients. The study included 104 patients, of whom 65 received targeted therapy first and 39 received radiotherapy first. The median progression-free survival was 11.1 months and 15.6 months, respectively, with no significant difference (P=0.096) 20. Moreover, there was no significant difference in OS (P=0.525), supporting the similar efficacy of targeted-first therapy or radiotherapy-first in EGFR mutation-positive lung cancer brain metastases patients 17. These results suggest that there is no significant impact on patient survival outcomes between targeted-first therapy and radiotherapy-first in EGFR mutation-positive lung cancer brain metastases patients. Similarly, our study results are consistent with these findings, with no significant difference observed between the two treatment sequences in terms of patient survival outcomes, indicating that targeted-first therapy may be a suitable treatment option for these patients from the perspective of protecting cognitive function.

The targeted-first therapy and radiotherapy-first treatment sequences show differences in local control, with radiotherapy-first having benefits for the local control of brain metastases. Studies have shown that there are some differences between the two treatment sequences in terms of local control, where radiotherapy-first has important benefits for controlling local brain metastases and targeted-first therapy may encounter some challenges in local control 14, 21. A relevant study explored the treatment strategies for EGFR mutation-positive lung cancer brain metastases. The results showed that radiotherapy-first can effectively control local brain metastases and arrest disease progression, while the local control effect of the targeted-first therapy group was poorer 21. The study's findings are consistent with ours. Another study obtained similar results 14. Therefore, in the treatment of EGFR mutation-positive lung cancer brain metastases, radiotherapy-first can achieve better local control by directly acting on brain metastases, which has the advantages of quickly relieving symptoms and improving neurological function. Subsequently, targeted therapy can be effective in controlling tumor spread and growth elsewhere when added to the regimen.

The targeted-first therapy and radiotherapy-first treatment sequences have not been shown to significantly differ in terms of iPFS. Most published studies have demonstrated differences between the two treatment sequences in terms of iPFS^22^. The study by Wang et al. also included iPFS information. Among all 74 patients in their study, 33 patients started targeted and radiotherapy-first simultaneously, 13 patients received initial radiotherapy-first followed by targeted therapy, and the remaining 28 patients received radiotherapy-first after targeted therapy failure. The iPFS for these three groups was 11.1 months, 11.3 months, and 8.1 months, respectively (P=0.032). However, our study results differed from these findings, possibly due to the small number of patients included. Collecting more patients and expanding the sample size may yield new results in the future.

### Limitations

The results of this study should be interpreted in the context of several limitations. Firstly, the study included a relatively small sample size of patients and lacked a standardized chemotherapy control cohort. Additionally, some patients were not regularly followed up and data collection was incomplete, so we need to interpret the data cautiously. Furthermore, relevant data on cognitive changes after treatment were lacking. Finally, relevant conclusions need to be further validated through prospective RCTs.

## Conclusion

Targeted-first regimen was not found to improve patients' iPFS relative to radiotherapy-first regimen in patients with brain metastases. Radiotherapy-first regimen for brain metastases demonstrated superior local control compared to targeted-first regimen. Patient's age, radiotherapy modality, metastasis volume, number of metastases, extracranial disease status and therapy sequence may be related to local control of metastases.

## Supplementary Material

Supplementary table.

## Figures and Tables

**Figure 2:**
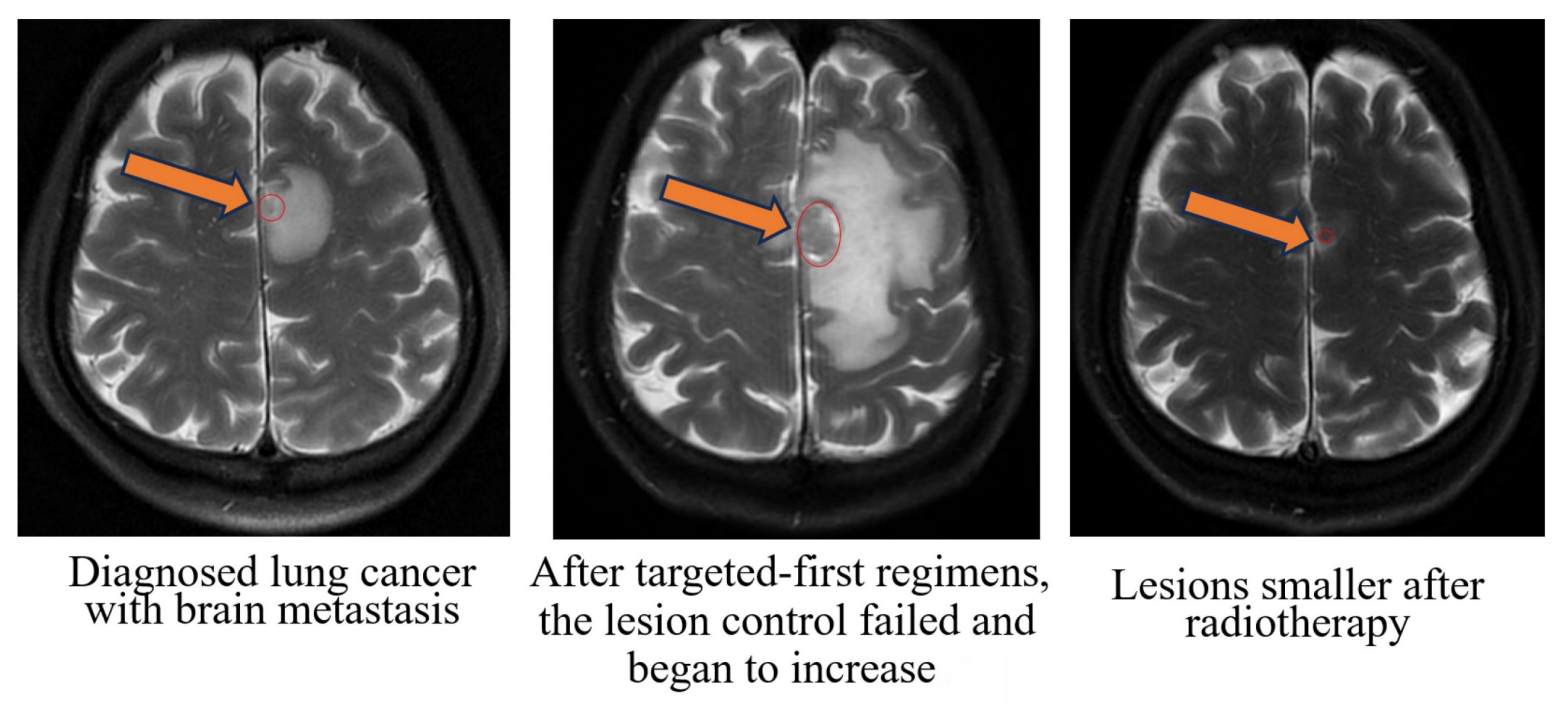
Radiographic images of a typical patient with gross tumor volume (GTV at the mark).

**Table 1:** Baseline clinical characteristics of patients with brain metastases.

Characteristic (88)	Targeted-first regimen (32)	Radiotherapy-first regimen (56)
**Age(years)**	(58.6±12.2)	(61.1±10.6)
<62	19(59.5%)	29(51.8%)
≥62	13(40.5%)	27(48.2%)
**Sex**		
Male	11(34.5%)	26(46.4%)
Female	21(65.5%)	30(53.6%)
**Extracranial disease status**		
Good	1(3.1%)	2(3.6%)
Bad (Uncontrolled)	31(96.9%)	54(96.4%)
**GTV volume(cm^3^)**	(9.1±14.6)	(13.5±14.9)
<5.6	18(56.3%)	21(37.5%)
≥5.6	14(43.8%)	35(62.5%)
**Radiotherapy modality (%)**		
SIB-WBRT	15(46.9%)	31(55.4%)
Brain metastases localized irradiation	17(53.1%)	25(44.6%)
**GTV number**		
>10	1(3.1%)	7(12.5%)
≤10	31(96.9%)	49(87.5%)

**Table 2:** Comparison of prognosis of patients in the radiotherapy-first regimen group versus the targeted-first regimen group (sample size = 88).

Ending	Number of ending events		HR	95% CI	*P*-value
	Targeted-first regimen	Radiotherapy-first (reference)			
Primary endpoint					
iPFS	15	29	1.180	0.622-2.237	0.613
Secondary endpoints					
OS	18	34	1.208	0.679-2.150	0.520
New Lesions	11	24	1.184	0.569-2.463	0.652
Local Control	23	64	2.397	1.453-3.954	<0.001

**Table 3:** Univariable and multivariable analysis of iPFS in patients with brain metastases.

Characteristics	Univariate analysis			Multivariate analysis	
	Hazard ratio(95%CI)	*P* value		Hazard ratio(95%CI)	*P* value
**Sex**		0.4450			0.2380
Male	Reference			Reference	
Female	0.790(0.431-1.447)			0.656(0.326-1.322)	
Age	0.998(0.970-1.027)	0.8910		1.003(0.971-1.035)	0.8720
**Therapy**		0.2020			0.0810
SIB-WBTRT	Reference			Reference	
Localized irradiation brain metastases	0.663(0.353-1.246)			0.528(0.258-1.082)	
**GTV volume**	1.005(0.985-1.026)	0.6250		0.988(0.776-1.284)	0.9880
**GTV number**		0.4430			0.4120
≤10	1.595(0.483-5.265)			0.582(0.160-2.120)	
>10	Reference			Reference	
**Extracranial disease status**		0.4710			0.3050
Bad (Uncontrolled)	0.586(0.137-2.508)			0.462(0.105-2.025)	
Good	Reference			Reference	
**GTV proportion**	/	/		/	/
**Therapy sequence**					
targeted-first	1.180(0.622-2.237)	0.6130		1.710(0.820-3.567)	0.1520
radiotherapy-first	Reference			Reference	

**Table 4:** Univariable and multivariable analysis of new lesions in patients with brain metastases.

Characteristics	Univariate analysis			Multivariate analysis	
	Hazard ratio(95%CI)	*P* value		Hazard ratio(95%CI)	*P* value
**Sex**		0.1330			0.1480
Male	Reference			Reference	
Female	0.597(0.305-1.169)			0.562(0.257-1.227)	
**Age**	0.992(0.959-1.026)	0.6390		0.997(0.961-1.035)	0.8860
**Therapy**		0.2260			0.0620
SIB-WBTRT	Reference			Reference	
Localized irradiation brain metastases	0.640(0.311-1.318)			0.451(0.195-1.042)	
**GTV volume**	0.992(0.961-1.024)	0.621		1.096(0.817-1.471)	0.539
**GTV number**		0.611			0.609
≤10	0.685(0.159-2.943)			0.667(0.142-3.145)	
>10	Reference			Reference	
**Extracranial disease status**		0.249			0.156
Bad (Uncontrolled)	0.421(0.098-1.837)			0.334(0.073-1.520)	
Good	Reference			Reference	
**GTV proportion**	/	/		/	/
**Therapy sequence**		0.652			0.209
Targeted-first	1.184(0.569-2.463)			1.733(0.735-4.085)	
Radiotherapy-first	Reference			Reference	

**Table 5:** Univariable and multivariable analysis of local control in patients with brain metastases.

Characteristics	Univariate analysis			Multivariate analysis	
	Hazard ratio(95%CI)	*P* value		Hazard ratio(95%CI)	*P* value
**Sex**		0.2820			0.5670
Male	Reference			Reference	
Female	0.781(0.497-1.226)			1.184(0.665-2.107)	
**Age**	1.056(1.030-1.082)	<0.001		1.054(1.026-1.082)	<0.001
**Therapy**		0.136			<0.001
SIB-WBTRT	Reference			Reference	
Localized irradiation brain metastases	1.467(0.887-2.426)			0.128(0.041-0.401)	
**GTV volume**	1.025(1.011-1.038)	<0.001		1.426(1.209-1.682)	<0.001
**GTV number**		<0.001			0.009
≤10	32.290(4.469-233.300)			14.960(1.990-112.444)	
>10	Reference			Reference	
**Extracranial disease status**		<0.001			0.023
Bad (Uncontrolled)	0.244(0.114-0.524)			0.387(0.170-0.880)	
Good	Reference			Reference	
**GTV proportion**	/	/		/	/
**Therapy sequence**		<0.001			<0.001
Targeted-first	2.397(1.453-3.954)			13.8(4.455-42.751)	
Radiotherapy-first	Reference			Reference	
