# Supplementary material for: Exploration of radiotherapy strategy for brain metastasis patients with driver gene positivity in lung cancer
Source: J Cancer. 2024 Feb 12;15(7):1994–2002. doi: 10.7150/jca.91875 (PMC10905398; doi:10.7150/jca.91875)
Supplement: Supplementary file 1 — Supplementary table. [file jcav15p1994s1.pdf]

Supplementary Table 6 Follow-up Results

Characteristics	Targeted-first **(32)**	Radiotherapy-first **(56)**
**iPFS**		
iPFS(%)	53.1(17/32)	48.2(27/56)
Time to iPFS Failure(range) (mo)	1.1-31.8	0.9-43.1
Median(mo)	13.4（95%: 6.860-20.000）	15.6（95%: 7.700-23.500）
iPFS at 6 months (%)	66.1（95%: 47.700-84.500）	87.1（95%: 77.300-96.900）
iPFS at 12 months (%)	45.1（95%: 23.700-66.500）	53.5（95%: 36.600-70.400）
**OS**		
OS（%）	43.75(14/32)	39.29(22/56)
Follow up time (months)	1.5-50.5	2.1-65.1
Median (range) (mo)	17.9（95%: 13.400-26.000）	23.5（95%: 14.700-32.300）
Overall Survival at 6 months (%)	80.3（95%:66.200-94.400）	85.5（95%:76.100-94.900）
Overall Survival at 12 months (%)	67.7（95%:49.900-85.500）	70.8（95%:58.300-83.300）
Overall Survival at 24 months (%)	28.7（95%:8.300-49.100）	46.4（95%:31.700-61.100）
**Local Control**		
Local Control (%)	76.0(73/96)	80.7(267/331)
Time to Local Failure(range) (mo)	1.2-13.4	0.9-43.1
Median (mo)	NR	23.8（95%: 15.2-32.5）
Local Control at 6 months (%)	73.6（95%:62.200-84.900）	94.9（95%:92.200-97.600）
Local Control at 12 months (%)	51.6（95%:34.400-68.800）	78.7（95%:72.000-85.400）
**New Lesion**		
New Lesion(s) (%)	34.38(11/32)	42.86(24/56)
Time to New Lesion(s) (range) (mo)	1.1-31.8	0.9-43.1
Median (mo)	17.4（95%: 2.400-32.400）	18.1（95%: 14.400-21.700）
No New Lesion & Survival at 6 months (%)	68.6（95%:50.200-87.000）	89.2（95%:80.200-98.200）
No New Lesion & Survival at 12 months (%)	60.1（95%:37.600-82.600）	66.5（95%:50.200-82.800）
